# Characterization of the complete chloroplast genome of Chinese rose, *Rosa chinensis* (Rosaceae: Rosa)

**DOI:** 10.1080/23802359.2019.1664350

**Published:** 2019-09-12

**Authors:** Wang Lin, Jing Huang, Meixiang Xue, Xiumei Wang, Chunhua Wang

**Affiliations:** Fujian Provincial Key Laboratory of Ecology-Toxicological Effects & Control for Emerging Contaminants, Key Laboratory of Ecological Environment and Information Atlas (Putian University) Fujian Provincial University, Key laboratory of Loquat Germplasm Innovation and Utilization (Putian University), Fujian Province University, College of Environmental and Biological Engineering, Putian University, Putian, China

**Keywords:** *Rosa chinensis*, Chinese rose, Rosaceae, complete chloroplast genome, phylogenetic analysis

## Abstract

*Rosa chinensis* known commonly as the Chinese rose, is a member of the genus Rosa native to Southwest China. In this study, the complete chloroplast genome of *R. chinensis* was sequenced and analyzed. Structural analysis of the complete chloroplast (cp) genome of *R. chinensis* that exhibits a typical quadripartite circular structure with 155,097 bp in size, which contains a large single-copy region (LSC) of 85,911 bp, a small single-copy region (SSC) of 17,270 bp and a pair of inverted repeat (IR) regions of 25,958 bp in each one. The cp genome of *R. chinensis* contains 130 genes, including 85 protein-coding genes, 37 tRNA genes and 8 rRNA genes. The phylogenetic Maximum-Likelihood (ML) analysis result shown that *R. chinensis* and *Rosa odorata* formed an independent clade with a 100% bootstrap support in phylogenetic relationship.

Chinese rose, *Rosa chinensis* is the most important ornamental plant with economic, cultural and symbolic value, which also is used as cut flowers as garden ornamental and for the perfume industry (Zhang and Zhu 2006). *Rosa chinensis* is the queen of flowers, and holds great symbolic and cultural value, which appeared as decoration on 5000 year-old Asian pottery (Wang 2007). Now, *R. chinensis* is greater economic importance than other flowers plants. It is widely cultivated around the world and sold as garden plants, in pots, or as cut flowers, the latter accounting for approximately 30% of the market (Nybom and Werlemark 2016). However, little information is available on the genome, genomics and molecular biology databases in Rosa. In this study, the complete chloroplast genome of *R. chinensis* is obtained and reported, which contributes to study the genetic diversity and the molecular breeding of the genus Rosa in the future.

The samples of *R. chinensis* were collected from flowers plantation in Putian district of Fujian province (Fujian, China, 119.07E; 25.49 N). The total cpDNA of *R. chinensis* form young leafs were extracted with the modified CTAB method and stored in Putian University (No. PTU004). The cpDNA was purified and fragmented using the NEB Next Ultra^TM^ II DNA Library Prep Kit (NEB, BJ, and CN) and was sequenced using the NGS method. Quality control was performed to remove low-quality reads and adapters using the FastQC (Andrews 2015). The chloroplast (cp) genome was assembled using the Plann (Huang and Cronk 2015) and annotated using the DOGMA (Wyman et al. 2004). The physical map of the chloroplast genome of *R. chinensis* was generated using OrganellarGenomeDRAW version 1.3.1 (Lohse et al. 2013). Here, the complete chloroplast genome sequence of *R. chinensis* was determined that the GenBank accession No. is MK8324431.

Structural analysis of the complete cp genome of *R. chinensis* that exhibits a typical quadripartite circular structure with 155,097 bp in size. It contains a large single-copy region (LSC) of 85,911 bp, a small single- copy region (SSC) of 17,270 bp and two inverted repeat regions (IRs) of 25,958 bp in each one. The overall nucleotide content of *R. chinensis* cp genome is: 31.0% of A, 31.8% of T, 18.9% of C, and 18.3% of G, with the total GC content is 37.2%. The cp genome of *R. chinensis* comprises 130 genes, which includes 85 protein-coding genes (PCGs), 37 transfer RNA (tRNA) genes and 8 ribosomal RNA (rRNA) genes. 18 genes were found duplicated in every one of the IR regions that includes 7 PCGs species (*rpl2*, *rpl23*, *ycf2*, *ndhB*, *rps7*, *rps12* and *ycf1*), 7 tRNAs species (*trnI-CAU, trnL-CAA, trnV-GAC, trnI-GAU, trnA-UGC, trnR-ACG* and *trnN-GUU*) and 4 rRNAs species (*rrn16, rrn23, rrn4.5* and *rrn5*).

To identify the phylogenetic position of *R. chinensis*, phylogenetic relationship analysis was conducted. The phylogenetic tree was reconstructed using Maximum-Likelihood (ML) methods and performed using the RaxML (Stamatakis 2014), which the bootstrap value was calculated using 5000 replicates to assess node support and all the nodes were inferred with strong support. The evolutionary tree was constructed and edited using the MEGA X (Kumar et al. 2018). The results indicated that *R. chinensis* is located in the genus Rose and is closest related to *R. odorata* (KF753637.1) (Figure 1). This study can continue in-depth the genetic diversity and the molecular breeding of the genus Rosa in future.

**Figure 1.**
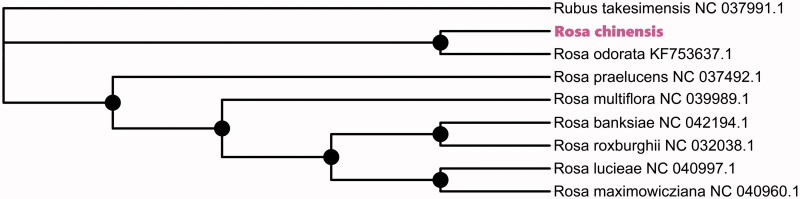
Phylogenetic relationship among 9 species based on the Maximum-Likelihood (ML) analysis of the complete chloroplast genome sequences from NCBI, using *Rubus takesimensis* as an outgroup. Bootstrap support values are indicated in each node.
